# Molecular diagnostics and inhibition of cross‐reactive carbohydrate determinants in Hymenoptera venom allergy

**DOI:** 10.1002/clt2.12230

**Published:** 2023-03-08

**Authors:** Dragana Jovanovic, Aleksandra Peric‐Popadic, Vojislav Djuric, Maja Stojanovic, Branislav Lekic, Ognjen Milicevic, Branka Bonaci‐Nikolic

**Affiliations:** ^1^ Clinic of Allergy and Immunology University Clinical Center of Serbia Belgrade Serbia; ^2^ University of Belgrade Faculty of Medicine Belgrade Serbia; ^3^ Clinic of Dermatovenerology University Clinical Center of Serbia Belgrade Serbia; ^4^ Department for Medical Statistics and Informatics Institute for Medicine Statistics and Informatics University Clinical Center of Serbia Belgrade Serbia

**Keywords:** carbohydrate determinants, honeybee venom, Hymenoptera, insect venom allergy, molecular diagnostics, wasps

## Abstract

**Background:**

The composition of venom extracts, cross‐reactive carbohydrate determinants (CCD) and the component‐resolved diagnostics (CRD) are important fields of investigation. IgE‐reactivity to CCD complicates the interpretation of IgE to Hymenoptera venoms, especially in patients with multiple‐positivity. We analyzed the clinical importance of CRD and CCD‐inhibition for selection of allergens for venom immunotherapy (VIT).

**Methods:**

In 71 patients, we measured specific IgE (sIgE) to honeybee venom (HBV), wasp venom (WV), hornet venom (HV), CCD, and recombinant allergens: phospholipase A2 (rApi m 1), hyaluronidase (rApi m 2), icarapin (rApi m 10), antigen 5 (rVes v 5), and phospholipase A1 (Immunoblot). In 29/71 HBV/WV/HV/CCD‐positive patients CCD‐inhibition was performed. According to CRD and CCD‐inhibition, we identified true sensitization and defined groups of multiple‐positive patients who needed CCD‐inhibition before starting VIT.

**Results:**

sIgE‐rApi m 1, sIgE‐rApi m 2, and sIgE‐rApi m 10 were detected in 65.7%, 68.4%, and 58%, respectively. In HBV allergic patients, CRD sensitivity was 86.8%. In WV allergic patients, sensitivity of sIgE‐rVes v 5 was 94%. True multiple‐sensitization was found in 44.8% of HBV/WV/HV/CCD‐positive patients after CCD‐inhibition. Patients with multiple venom‐ and CCD‐positivity had more frequent severe allergic reactions (*p* < 0.001). CCD‐inhibition was helpful in HBV/WV/HV/CCD‐positive patients who were negative to all tested recombinant honeybee allergens. Persistence of HBV‐positivity after CCD‐inhibition requires CRD to other honeybee recombinant allergens.

**Conclusion:**

CRD, using a profile of five most important recombinant allergens and CCD, has a high sensitivity for the diagnosis of venom allergy, especially in patients positive to several venom extracts. CRD and CCD‐inhibition are helpful to reveal the clinically relevant, true sensitization and improve the selection of venoms for long‐lasting VIT.

## 1 BACKGROUND

Hymenoptera venoms are known to cause life‐threatening and fatal immunoglobulin E (IgE)‐mediated anaphylactic reactions. About 50%–60% of patients with Hymenoptera venom allergy (HVA) have specific IgE (sIgE) reactivity to both insect–species, honeybee (*Apis mellifera*) and yellow jacket (*Vespula vulgaris*, also called wasp) or hornet (*Vespa crabro*). Protein‐based and carbohydrate determinants‐based cross‐reactivity are the most frequent causes of multiple‐positivity.^1^

Venom extracts are complex, heterogeneous mixtures rich in glycoproteins. Cross‐reactive carbohydrate determinants (CCD), composed of α‐1,3‐fucosylated *N*‐glycans, interfere with the detection of clinically relevant IgE specific to protein epitopes.^2^ In contrast to diagnostics based on whole venom extracts, the component‐resolved diagnostics (CRD) reveal sIgE to single recombinant allergens without CCD. In patients positive to several venom extracts, CRD can distinguish true, clinically relevant IgE sensitization to protein epitopes from sIgE‐reactivity to CCD. Compared to diagnostics based on whole venom extracts, CRD improves the selection of allergens that will be used for long‐lasting venom immunotherapy (VIT).^3^, ^4^, ^5^

Hymenoptera proteins involved in cross‐reactivity are hyaluronidases (Api m 2 and Ves v 2) and dipeptidyl‐peptidases (Api m 5 and Ves v 3) from honeybee and wasp, respectively.^2^ Cross‐reactivity caused by CCD can be eliminated using CRD and CCD‐inhibition that are underused in clinical practice.^5^ False‐positive results are a common problem in the *in vitro* diagnostics of HVA.^6^ The majority of venom allergens are glycoproteins and cross‐linking of only CCD epitopes with mast cell‐bound IgE does not lead to mast cell degranulation. sIgE‐reactivity to whole venom extract does not allow a distinction between sIgE‐reactivity to protein epitopes from sIgE‐reactivity to CCD epitopes.^7^, ^8^ Extract‐based diagnostics limit the use of standard sIgE serology for selection of causative venom for VIT. In some cases, despite finding of sIgE, patients did not have allergy symptoms, because sensitization was caused by well‐tolerated venom allergen. VIT over 3–5 years with insect venom is still the only effective and causal treatment of HVA. The clinical importance of sIgE against CCD epitopes (CCD‐sIgE) is controversial, although most studies showed that CCD‐sIgE have no clinical relevance.^9^, ^10^, ^11^ Due to the clinically irrelevant CCD‐sIgE, increased levels of sIgE to conventional whole venom extract should be interpreted carefully, in the context of the clinical history. Unfortunately, identification of the allergy‐relevant venom is sometimes difficult, because the patient could not identify the insect.^4^ Moreover, some patients with high sIgE‐levels do not show clinical manifestations, while other patients with low sIgE‐levels may experience life‐threatening systemic allergic reactions (SAR).^4^, ^8^

In our previous study, we showed that CCD‐sIgE were more frequently found in honeybee allergic patients.^4^ This finding can be explained by the fact that majority of honeybee allergens are glycosylated, while the two major wasp allergens are not glycosylated.^12^, ^13^ Also, we found that the use of the major allergen rApi m 1 (phospholipase A2) was not sufficient, regardless of the detection method. On the other hand, using recombinant major wasp allergen rVes v 5 (antigen 5) was enough for about 90% of wasp allergic patients.^4^

The variability of test systems in allergy diagnostics is due to a lack of international standards for allergens and antibodies. This often leads to different test results and complicates their comparability.

This study aimed to analyze the diagnostic importance of CRD for simultaneous determination of five most important recombinant allergens and CCD‐inhibition in patients with HVA. According to the reliable history of insect sting allergy, and the sIgE‐test we intended to identify criteria for true sensitization and to define groups of multiple‐positive patients who needed CCD‐inhibition before starting long‐term VIT.

## 2 METHODS

### 2.1 Patients

This retrospective, single‐center study included 71 Hymenoptera allergic patients: 46 male (64.8%) and 25 female (35.2%) (mean age 46.3 ± 15.3 years, range 18–75 years) with sIgE ≥0.35 kUA/L who have been followed‐up as outpatients or in‐patients from November 2019 to January 2022, at the Clinic of Allergy and Immunology, University Clinical Center of Serbia in Belgrade. A blood sample for analysis was taken from all patients who agreed to participate in the study signing the informed consent. The study has been approved by the Ethics Committee of the Medical Faculty, University of Belgrade, and number 1550/V‐8.

Patients who had a documented history of a reliable culprit insect that caused SAR were included in the study. We classified our patients in two groups: CCD‐sIgE positive (29/71) and CCD‐sIgE negative (42/71). Severity of SAR to Hymenoptera stings were classified according to Mueller ranging from I to IV degree (Table 1).^14^

**TABLE 1:** Comparison of demographic characteristics and degree of severity systemic allergic reaction in 71 patients with Hymenoptera venom allergy, with and without CCD‐sIgE.

	CCD‐sIgE negative (*n* = 42)	CCD‐sIgE positive (*n* = 29)
Age (years)	43.6 ± 15.3	50.1 ± 14.8
Female (%)	31 (73.8)	15 (51.7)
Male (%)	11 (26.2)	14 (48.3)
Honeybee (%)	20 (47.6)	12 (41.3)
Wasp (%)	20 (47.9)	8 (27.6)
Hornet (%)	2 (4.7)	3 (10.3)
Honeybee and wasp (%)	0	5 (17.2)
Honeybee and hornet (%)	0	1 (3.4)
Degree of severity sting reactions (%)		
I	12 (28.6)	0
II	2 (4.8)	2 (6.8)
III	16 (38.1)	14 (48.3)
IV	12 (28.6)	13 (44.8)

Abbreviations: CCD, cross‐reactive carbohydrate determinants; sIgE, specific IgE.

### 2.2 Methods

Serum sIgE‐levels to honeybee venom (HBV), wasp venom (WV), hornet venom (HV), and recombinant allergens: rApi m 1, rApi m 2, icarapin (rApi m 10) from HBV, rVes v 5, phospholipase A1 (rVes v 1) from WV, and CCD were measured by Immunoblot (Euroimmun, Germany, Euroline DPA‐Dx insect venoms 3). Serum samples of 29/71 multiple‐positive patients were tested in the EUROLINE assay using the anti‐CCD Absorbent (Euroimmun). The absorbent (CCD‐inhibitor) for eliminating CCD‐sIgE was composed of glycoprotein bromelain extracted from pineapple. Serum samples were incubated with anti‐CCD absorbent for 60 min at room temperature according to the manufacturer's instructions. The sera were retested using the same insect venoms profile, as previously mentioned.

The cut‐off value for positive sIgE‐test results was 0.35 kUA/L. The sIgE‐reactivity was categorized quantitatively into six classes: Class 1 (≥0.35 to <0.70 kUA/L), Class 2 (≥0.70 to <3.50 kUA/L), Class 3 (≥3.50 to <17.50 kUA/L), Class 4 (≥17.50 to <50.00 kUA/L), Class 5 (≥50.00 to <100.00 kUA/L), and Class 6 (≥100.00 kUA/L).

### 2.3 Statistical analyses

Obtained data were analyzed using IBM SPSS Statistics software for Windows (version 17; IBM, Armonk, NY). Mean quantitative variables were used and frequency of qualitative variables were also calculated. Nonparametric Chi‐square and Fisher exact tests were used to evaluate the relationship between variables. The Wilcoxon signed‐rank test and *t*‐test were used to compare two matched samples. According to a history of insect sting allergy, we calculated sensitivity using the formula: Sensitivity = true‐positive/true‐positive + false‐negative ×  100%.

## 3 RESULTS

A comparison of demographic characteristics of CCD‐sIgE negative (42/71) and CCD‐sIgE positive patients (29/71) is shown in Table 1.

The majority of patients (55/71) had severe SAR (III and IV grades of SAR) (77.5%) (*p* < 0.001). No significant differences were found in the frequency of severe SAR between female and male patients.

In the CCD‐sIgE positive group, more patients had severe SAR to Hymenoptera sting 27/29 (93%) than mild SAR 2/29 (6.9%) (*p <* 0. 001).

Among the 71 patients, 39 (55%) patients showed multiple‐positivity, while 30 (42.3%) showed single‐positivity: 13 to HBV extract, 15 to WV extract, and 2 to HV extract. Only two (2.8%) patients with a history of SAR after a wasp stings did not show sIgE‐reactivity to venom extracts (Table 2). A significant number (29/39) of patients had multiple‐ and CCD‐positivity (HBV/WV/HV/CCD‐positive). The rest of the patients (10/39) had multiple‐positivity (HBV/WV or HBV/WV/HV positive) but without CCD‐sIgE‐positivity (Tables 2 and 3).

**TABLE 2:** Specific IgE to recombinant honeybee and wasp allergens in 42 Hymenoptera venom allergic patients without CCD‐sIgE.

IgE (immunoblot)	Multiple‐positivity to HBV/WV/HV or HBV/WV extracts (*n* = 10)	Single‐positivity to HBV extract (*n* = 13)	Single‐positivity to WV extract (*n* = 15)	Single‐positivity to HV extract (*n* = 2)	Negative for HBV and WV and HV extract (*n* = 2)
rApi m 1 + and/or rApi m 2 + and/or rApi m 10 + and rVes v 5 + and rVes v 1 +/–	5 (50%)	0	0	0	0
rApi m 2 + and rVes v 5 + rVes v 1 +/–	0	0	0	0	1 (50%)
rApi m 1 + and/or rApi m 2 + and/or rApi m 10 +	4 (40%)	9 (69.2%)	0	0	0
rApi m 2 + only	0	2 (15.4%)	0	0	0
rVes v 5 + and rVes v 1 +/–	1 (10%)	0	13 (86.6%)	2 (100%)	1 (50%)
rApi m 1 – rApi m 2 – rApi m 10 – rVes v 5 – r rVes v 1 –	0	2 (15.4%)	2 (13.3%)	0	0

Abbreviations: CCD, cross‐reactive carbohydrate determinants; HBV, honeybee venom; HV, hornet venom; rApi m 1, recombinant phospholipase A2; rApi m 2, recombinant hyaluronidase; rApi m 10, recombinant icarapin; rVes v 1, recombinant phospholipase A1; rVes v 5, recombinant antigen 5; WV, wasp venom.

**TABLE 3:** Levels of specific IgE (Class I‐VI) to venom extracts and recombinant honeybee and wasp allergens in 29 multiple HBV/WV/HV/CCD‐positive Hymenoptera venom allergic patients, before and after CCD‐inhibition.

Insect	CCD‐sIgE positive patients before CCD‐inhibition								CCD‐sIgE positive patients after CCD‐inhibition							
	HBV	WV	HV	rApi m 1	rApi m 2	rApi m 10	rVes v 5	rVes v 1	HBV	WV	HV	rApi m 1	rApi m 2	rApi m 10	rVes v 5	rVes v 1
I																
HB/W	4	5	4	2	3	2	6	1	3	5	3	4	4	3	6	1
HB/W	5	2	2	2	3	0	2	0	5	1	1	2	4	0	2	0
HB/W	5	4	4	0	1	2	1	2	3	4	0	1	1	3	1	2
HB/W	4	2	3	3	2	0	2	0	2	2	1	4	2	0	2	0
W	2	2	2	1	0	0	3	0	2	2	1	2	0	0	3	0
HB/H	5	4	5	0	5	2	5	1	4	4	4	0	5	2	5	0
HB/W	3	1	1	1	4	3	3	0	3	1	1	2	4	3	3	0
II																
H	4	3	4	0	5	0	4	0	4	3	3	0	5	0	3	0
H	3	3	3	0	5	0	3	1	3	3	3	1	5	0	3	0
W	1	1	1	0	1	0	1	0	1	1	1	0	1	0	1	0
W	3	3	4	0	3	0	4	0	1	3	4	0	3	0	4	0
III																
HB	5	3	5	4	6	4	0	0	5	3	3	5	6	5	0	0
HB	4	2	2	2	5	3	0	0	4	0	0	3	5	4	0	0
HB	4	1	2	3	5	4	0	0	5	0	0	4	5	5	0	0
HB	2	1	1	4	2	1	0	0	3	0	0	5	2	1	0	0
HB	4	1	1	5	0	6	0	0	5	0	1	5	0	6	0	0
HB	4	1	2	4	0	4	0	0	3	0	0	4	0	4	0	0
HB	5	3	4	5	5	4	0	0	5	0	0	6	5	4	0	0
HB	3	1	1	0	2	3	0	0	2	0	0	0	2	3	0	0
HB	5	3	3	5	2	5	0	0	5	0	0	5	3	5	0	0
IV																
HB	4	3	4	0	0	0	0	0	2	1	0	1	0	0	0	0
HB	3	1	2	0	0	0	0	0	3	1	1	0	0	0	0	0
HB	3	2	3	0	0	0	0	0	2	1	0	0	1	0	0	0
V																
W	1	2	1	0	0	0	3	0	1	2	1	0	1	0	3	0
W	3	3	3	0	0	0	4	1	1	2	2	0	0	0	3	1
H	2	3	3	0	0	0	2	0	3	3	3	0	0	0	2	0
W	3	2	2	0	0	0	5	0	2	2	1	1	0	1	4	0
W	3	3	3	0	0	0	4	0	3	3	2	0	0	0	3	0
W	2	2	2	0	0	0	3	0	1	2	1	0	0	0	3	0

*Note*: Shaded areas: Levels of IgE (Class I‐VI) to recombinant honeybee and wasp allergens.

Abbreviations: CCD, cross‐reactive carbohydrate determinants; H, hornet; HB, honeybee; HBV, honeybee venom; HV, hornet venom; rApi m 1, recombinant phospholipase A2; rApi m 2, recombinant hyaluronidase; rApi m 10, recombinant icarapin; rVes v 1, recombinant phospholipase A1; rVes v 5, recombinant antigen 5; sIgE, specific IgE; W, wasp; WV, wasp venom.

The majority of patients (65/71) experienced SAR after one insect sting. In all single‐positive patients, a history of insect sting allergy agreed with the finding of sIgE to the corresponding venom extract. Among patients with multiple‐positivity, 19/39 had SAR to the honeybee, 11/39 to wasp, 3/39 to hornet, while 6/39 had two separate SAR to the honeybee and wasp or honeybee and hornet stings.

Among the wasp allergic patients, 31/33 (94%) patients had sIgE‐reactivity to rVes v 5, while 6/31 (19.4%) had sIgE‐reactivity to rVes v 5 and rVes v 1 (*p* < 0.001).

All six hornet allergic patients had sIgE‐reactivity to rVes v 5, while 2/6 (33.3%) of them had sIgE‐reactivity to rVes v 5 and rVes v 1. There were no wasp or hornet allergic patients who had an isolated sIgE‐reactivity to rVes v 1 (Tables 2 and 3).

**FIGURE 1:**
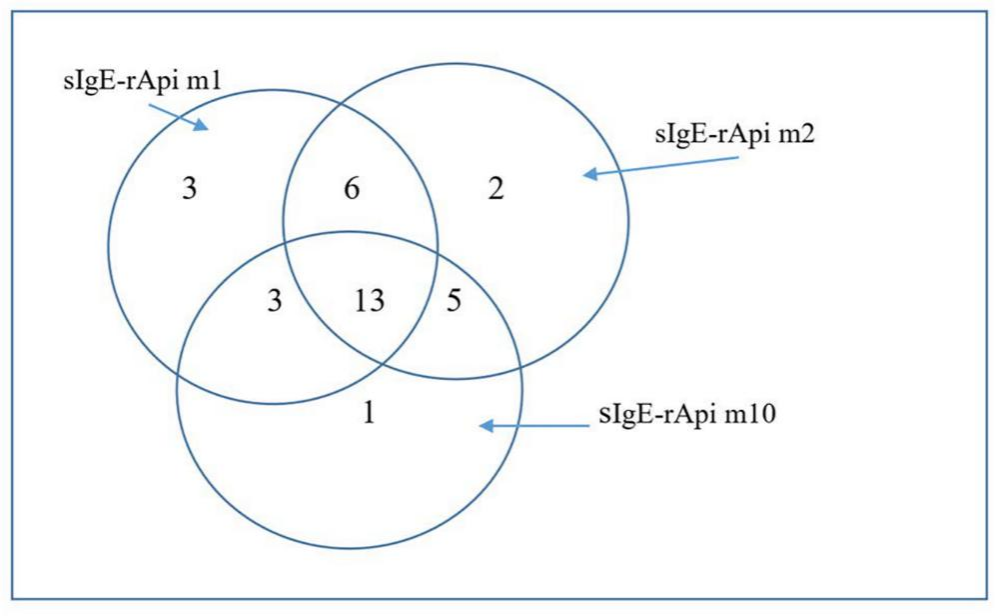
Specific IgE (sIgE) profile of recombinant major honeybee allergens in 33/38 patients with honeybee venom allergy. rApi m 1, recombinant phospholipase A2; rApi m 2, recombinant hyaluronidase; rApi m 10, recombinant icarapin; sIgE, specific IgE.

Using Immunoblot analysis, 33/38 (86.8%) honeybee allergic patients presented HBV‐positivity and all 33 patients had sIgE‐reactivity to at least one tested recombinant honeybee allergen. By analyzing HBV‐positive sera, 13/38 (31.6%) were sIgE‐reactive to all tested recombinant honeybee allergens, 25 (65.7%) to rApi m 1, 26 (68.4%) to rApi m 2, and 22 (57.9%) to rApi m 10 (Figure 1). A small number 5/38 (13.2%) of patients had no IgE‐reactivity to any of the tested recombinant honeybee allergens.

True multiple‐sensitization to both insect–species determined by sIgE‐reactivity to at least one recombinant honeybee allergen and rVes v 5 were found in 16/39 (41%) multiple‐positive patients and in none of the 30 single‐positive patients (*p* < 0.001). Seven of 71 (9.9%) patients were negative for all tested recombinant allergens (Tables 2 and 3).

According to CRD and CCD‐inhibition, five different subgroups of HBV/WV/HV/CCD‐positive patients were distinguished (Table 3).

In the first group of selected patients (7/29) with a history of SAR to honeybee and wasp/hornet stings (except for one patient who experienced SAR due to wasp sting), we found a true multiple‐positivity and a significant decrease of HV‐positivity (*p* < 0.05) after CCD‐inhibition.

The second group (4/29) of patients with a history of SAR due to wasp/hornet sting had sIgE‐reactivity to rApi m 2 and rVes v 5, before and after CCD‐inhibition.

In the third group of selected patients (9/29) with a history of SAR to honeybee sting, IgE‐testing confirmed a honeybee allergy due to sIgE‐reactivity to the majority of tested recombinant honeybee allergens and loss of WV‐positivity (*p* < 0.01) and HV‐positivity (*p* < 0.05) after CCD‐inhibition. We showed that CCD‐inhibition significantly increased sensitivity for HBV‐single‐positivity from 0% to 77%.

In the fourth group of selected patients (3/29) with a history of SAR to honeybee sting, there was no sIgE‐reactivity to all tested recombinant allergens. After CCD‐inhibition, in 2/3 patients we found sIgE‐reactivity to rApi m 1 or rApi m 2.

The fifth group of selected patients (6/29) with a history of SAR to wasp/hornet stings, showed initial sIgE‐reactivity only to rVes v 5. After CCD‐inhibition, 2/6 patients showed sIgE‐reactivity to rApi m 1 and rApi m 10 or rApi m 2, in addition to sIgE‐reactivity to rVes v 5.

In the group of 29 HBV/WV/HV/CCD‐positive patients, all patients completely lost CCD‐positivity after CCD‐inhibition (*p* < 0.01). 22/29 patients retained HBV/WV‐ or HBV/WV/HV‐positivity, but a significant decrease was found in the mean concentration of sIgE to WV and HV extracts (Table 4). A significant decrease was found in the mean concentration of sIgE to rVes v 5. On the contrary, a substantial increase was found in the mean concentration of sIgE to rApi m 1 and rApi m 2 after CCD‐inhibition (Table 4).

**TABLE 4:** Levels of specific IgE to venom extracts and recombinant allergens in 29 multiple HBV/WV/HV/CCD‐postive Hymenoptera venom allergic patients, before and after CCD‐inhibition.

sIgE (Immunoblot) kUA/l	Before CCD‐inhibition	After CCD‐inhibition	*P*
HBV extract (mean ± SD)	41.01 ± 22.02	32.93 ± 25.46	0.066
WV extract (mean ± SD)	13.58 ± 18.47	11.31 ± 17.82	0.047
HV extract (mean ± SD)	31.72 ± 28.37	18.63 ± 13.35	0.001
rApi m 1 (mean ± SD)	14.62 ± 25.29	27.39 ± 37.73	0.012
rApi m 2 (mean ± SD)	41.67 ± 43.83	46.09 ± 45.58	0.031
rApi m 10 (mean ± SD)	17.11 ± 34.34	25.79 ± 41.07	0.076
rVes v 5 (mean ± SD)	28.05 ± 35.33	20.81 ± 31.57	0.004
rVes v 1 (mean ± SD)	0.22 ± 3.12	0.19 ± 3.0.31	0.502

Abbreviations: CCD, cross‐reactive carbohydrate determinant; HBV, honeybee venom; HV, hornet venom; rApi m 1, recombinant phospholipase A2; rApi m 2, recombinant hyaluronidase; rApi m 10, recombinant icarapin; rVes v 1, recombinant phospholipase A1; rVes v 5, recombinant antigen 5; sIgE, specific IgE; WV, wasp venom.

**FIGURE 2:**
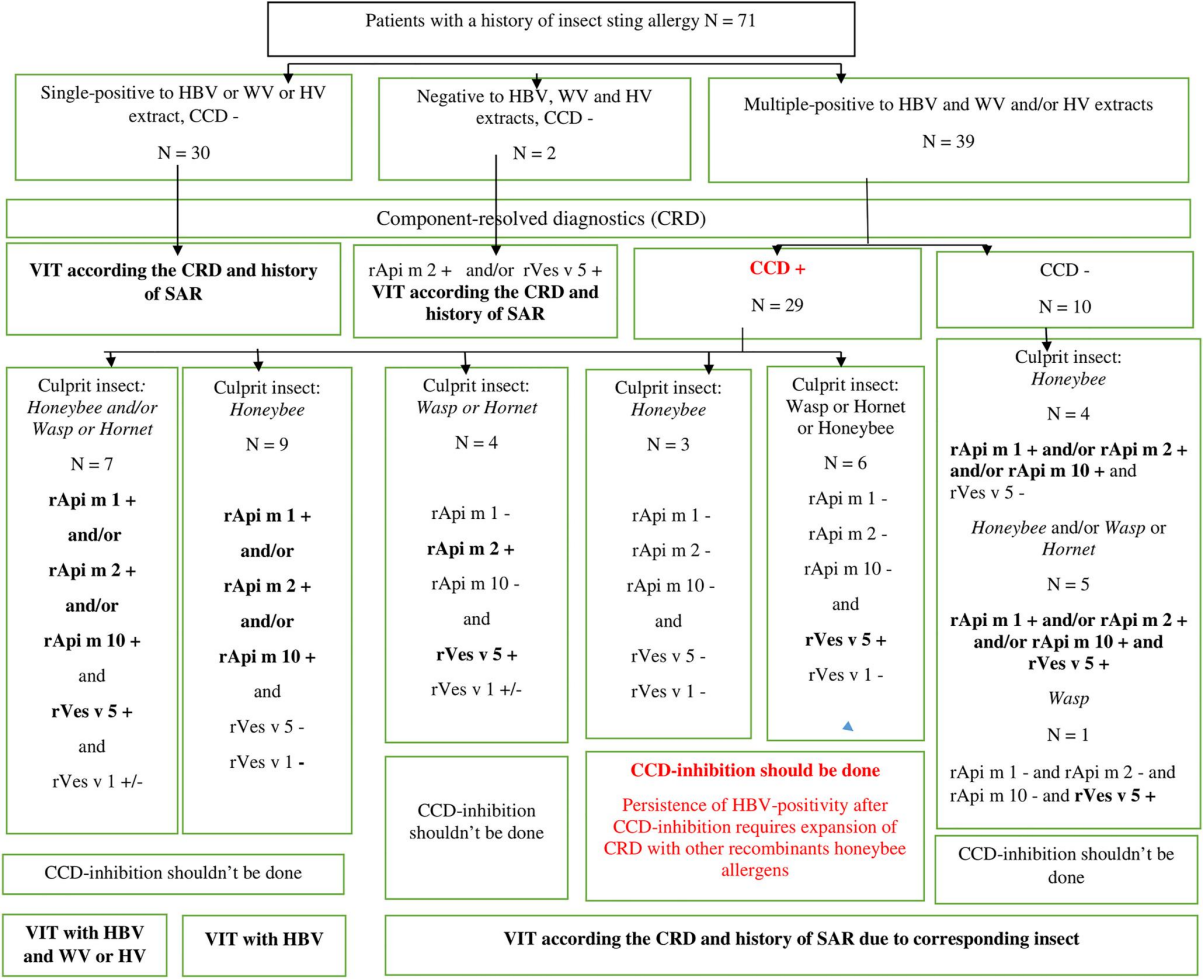
Algorithm for the necessary diagnostic steps that ensure an adequate therapeutic approach for long‐lasting VIT in 71 patients with Hymenoptera venom allergy. CCD, cross‐reactive carbohydrate determinants; CRD, component‐resolved diagnostics; HBV, honeybee venom; HV, hornet venom; rApi m 1, recombinant phospholipase A2; rApi m 10, recombinant icarapin; rApi m 2, recombinant hyaluronidase; rVes v 1, recombinant phospholipase A1; rVes v 5, recombinant antigen 5; VIT, venom‐immunotherapy; WV, wasp venom.

According to our results, we proposed an algorithm for the necessary diagnostic steps to ensure an adequate therapeutic approach for long‐lasting VIT (Figure 2).

## 4 DISCUSSION

Our study demonstrates the clinically importance of molecular CRD, using profile of the five most important recombinant allergens and CCD‐inhibition of CCD‐sIgE‐positive sera in the daily clinical practice. CRD and CCD‐inhibition were helpful in determining true sensitization in our multiple‐positive patients who had HVA. This diagnostic approach should be extremely important for the selection of adequate venom for long‐lasting VIT.^5^, ^15^ For the first time we identified different clusters of patients that were classified in the five subgroups of HBV/WV/HV/CCD‐positive patients, sharing the characteristic patterns of a history of insect sting allergy and serological sIgE‐tests.

Our previously published study showed that we must increase the number of recombinant honeybee allergens, tested by Immunoblot.^4^ We especially analyzed a group of patients with multiple‐positivity in order to determine how CCD‐inhibition affects sIgE‐results and whether it is necessary to apply CCD‐inhibition in all CCD‐sIgE‐positive patients.

We found that 39/71 patients had multiple‐positivity, while 29/39 patients had CCD‐sIgE. More recent studies revealed that much of the cross‐reactivity between wasp and honeybee allergens is not due to protein cross‐reactivity but is caused by CCD.^16^, ^17^, ^18^

α‐1,3‐Linked fucose residue on the *N*‐acetyl‐glucosamine *N*‐glycan are present in phospholipase A2 (Api m 1) from HBV, and hyaluronidases (Api m 2, Ves v 2) from HBV and WV, respectively,^19^, ^20^, ^21^ and these epitopes can induce the production of anti‐CCD antibodies of different isotypes.^22^, ^23^

Our HBV/WV/HV/CCD‐positive patients (27/29) had more often severe SAR (*p* < 0.001). Although, we could not rule out the possibility that patients with mild SAR were less frequently present in our group, it has been previously shown that glycosylation can contribute to epitope availability, immunoreactivity, and enzymatic function of native allergens.^8^, ^22^, ^24^

CCD‐sIgE have been reported to be responsible for more than 50% of double‐sensitizations to HBV and WV.^25^, ^26^ Both, previously studied data and our data showed that about half of the patients with HVA develop CCD‐sIgE.^4^, ^26^ CCD‐sIgE represent a pitfall of *in vitro* diagnostics because they cause multiple‐reactivity with any glycosylated allergens (pollen, food, or venom) and significantly interfere with the detection of clinically relevant IgE‐sensitization to protein epitopes.^5^, ^27^ The double‐ or triple‐positivity of sIgE to whole venom complicates the choice of appropriate VIT, resulting in increased risk of side effects, de novo sensitizations and higher costs.^19^ In these patients, CRD and CCD‐inhibition provide more accurate diagnostic results.^7^, ^17^

In most countries, the accepted clinical standards recommend skin‐testing as the initial method.^28^ However, skin‐testing may show inconclusive multiple‐positive results that further complicate the diagnosis of HVA. The significance of *in vitro* diagnosis of allergy indicates the importance of CCD‐inhibition because it eliminates false‐positive results.^17^

We demonstrated that CCD‐inhibition should not be performed in all CCD‐sIgE‐positive patients. Based on CRD and CCD‐inhibition, we found the agreement between the results of the IgE‐testing and the history of insect sting allergy.

The decision process for VIT is very important for the efficiency of the therapy, and in most cases treatment with only one venom appears to be sufficient. Among the HBV/WV/HV/CCD‐positive patients with wasp/hornet allergy, 5/29 patients after CCD‐inhibition showed sIgE‐reactivity to rApi m 2 and rVes v 5. Although, Api m 2 shows a high percentage of homology with Ves v 2,^27^, ^29^ it can sometimes be difficult to determine whether these results reflect sensitization due to cross‐reactivity between the shared protein determinants or true double sensitization to both insect species. Since HBV‐allergic patients can have Api m 2 mono‐sensitization, a decision regarding VIT in patients with IgE‐reactivity to rApi m 2 and rVes v 5 should be based on CRD, severity of SAR and history of insect sting allergy.^27^, ^30^, ^31^

Interestingly, the sensitivity of HBV‐single‐positivity was significantly increased after CCD‐inhibition from 0% to 77% in our third group of HBV/WV/HV/CCD‐positive honeybee allergic patients. In this group of patients CCD‐inhibition can replace expensive CRD.

We demonstrated that CCD‐inhibition is especially helpful in the subgroup of HBV/WV/HV/CCD‐positive patients who were negative for all tested recombinant allergens and in the subgroup of HBV/WV/HV/CCD/rVes v 5‐positive patients. In these patients, HBV‐positivity persisted after CCD‐inhibition. Therefore, these patients require an extended CRD to other recombinant honeybee allergens. Also, the number of patients with sIgE‐reactivity to recombinant honeybee allergens increased after CCD‐inhibition.

Having in mind that sIgE‐sensitization can be found incidentally in a patient without a clinical history of SAR, the possibility of an allergic reaction to a future insect sting cannot be completely excluded. Due to the possibility of an adverse effects on a future sting, and according to the latest VIT guidelines of the European Academy of Allergy and Clinical Immunology (EAACI), it is not wrong to perform VIT with two venoms in patients with true double‐positivity according to results of CRD and CCD‐inhibition.^32^

In this context, the newly detected sIgE‐reactivity to recombinant honeybee allergens in our four venom‐allergic patients after CCD‐inhibition was very important. It is already known, that sIgE‐levels do not correlate with the severity of the allergic reaction.^4^, ^33^ Also, long‐lasting VIT should be considered for patients with honeybee sensitization because they are at a higher risk for SAR.^32^

Surprisingly, we are the first to observe a significant increase of sIgE‐levels to rApi m 1 and rApi m 2 (*p* < 0.05), and a significant decrease of sIgE‐levels to rVes v 5 (*p* < 0.01) after CCD‐inhibition in HBV/WV/HV/CCD‐positive patients. The exact reasons for changes of sIgE‐levels to recombinant allergens after CCD‐inhibition are not entirely clear. In fact, this finding may be caused by different structure of native HBV and WV allergens and by difficulties in obtaining purified recombinant allergens. Unfortunately, the recombinant proteins can be modified by posttranslational modifications in Golgi apparatus of eukaryotic cells and may bind different sugars.^34^ In allergic patients, native glycosylated honeybee proteins trigger production of CCD‐sIgE, but also CCD‐sIgG (all subclasses) of different affinities.^35^, ^36^ Our results indirectly indicate that rApi m 1 and rApi m 2 contain small amounts of CCD antigens, which may allow CCD‐sIgG and CCD‐sIgE to interfere with the binding of sIgE to protein epitopes. This produces falsely lower sIgE‐levels before CCD‐inhibition. Finally, after CCD‐inhibition, protein sIgE epitopes are more accessible, leading to true, higher sIgE‐levels to rApi m 1 and rApi m 2. This hypothesis can also be an explanation for the increased number of patients with sIgE to recombinant honeybee allergens after CCD‐inhibition.

Contrary, Ves v 5 is not naturally glycosylated and can only induce the production of protein‐based antibodies. But, we suppose that rVes v 5 in Immunoblot contains small amounts of CCD antigens, which allows binding of ubiquitous CCD‐sIgE. Constant contact with food‐allergens in the gastrointestinal tract and pollen (glycosylated proteins) on the mucosal surfaces of the respiratory system, can induce production of certain sIgE‐levels against the CCD epitopes.^35^ This leads to false, higher sIgE‐levels to rVes v 5 before CCD‐inhibition. CCD‐inhibition reduces non‐specific binding of CCD‐sIgE leading to true, lower sIgE‐levels to rVes v 5.

Our results indicated that CCD‐inhibition can overcome current limitations of some procedures in recombinant technology, especially in Immunoblot which do not display three‐dimensional conformational structure of antigens that hide or change presentation of some IgE epitopes.^37^ Nevertheless, the recombinant production of certain allergens still needs to overcome technological hurdles and the development of purified CCD‐free, correctly folded recombinant proteins is mandatory in allergy diagnostics and immunotherapy.

In this study with a larger number of honeybee allergic patients, the sensitivity of Immunoblot for rApi m 1 was 65.7%. Similarly, we detected solid sensitivity for rApi m 2 and rApi m 10 (68.4% and 58%, respectively) in honeybee allergic patients. Sensitivity of CRD using simultaneous detection of sIgE for at least one tested recombinant honeybee allergen was 86.8%. So far, 12 HBV allergens have been described until now, and Api m 1 and Api m 4 (melittin) are found in significant amounts in HBV.^36^ One study showed that Api m 2 and Api m 10 are important major allergens with sIgE‐reactivity in the range of 47.9%–52.2% and 61.8%–72.2%, respectively.^38^

Importantly, our result identified a very high sensitivity for rVes v 5 in wasp allergic patients (94%) and hornet allergic patients (100%). We also demonstrated no patients with wasp or hornet allergy who had only sIgE‐reactivity to rVes v 1. The minority of patients with wasp and hornet allergy (8/39) had sIgE‐reactivity to rVes v 5 and rVes v 1. The results of several studies have confirmed that rVes v 5 is sufficient in most patients for serological confirmation of wasp or hornet allergy.^15^, ^26^, ^39^ When a differential diagnosis between wasp and hornet allergy is required a history of insect sting allergy is very important, in addition of sIgE‐reactivity to rVes v 5. In addition, the sensitization rate of honeybee allergic patients to rApi m 1 is 57% to 83.3%, and sensitization rate of wasp allergic patients to rVes v 5 is 86.55%–96.7% measured by ImmunoCAP.^38^, ^40^, ^41^ In line with these observations, our results confirmed that Immunoblot had satisfactory sensitivity to the same allergens.

Since, CRD in our study failed to diagnose (5/38) 13.2% of honeybee allergic patients, and (2/33) 6.1% of wasp allergic patients, it may suggest that additional CRD are needed in these patients. On the other hand, CRD can help identify patients with a history of insect sting allergy without sIgE‐reactivity to venom extracts,^42^ as it was in two of our wasp allergic patients.

Although, sIgE‐levels during successful VIT do not correlate with clinical improvement, changes in IgE‐ and IgG4‐levels are known to indicate VIT‐induced improvements in immune tolerance.^19^, ^43^ Since the IgG/IgE ratio is a good marker of efficacy,^44^ it is important to know their true initial concentrations, before starting long‐term VIT. Future studies will elucidate whether sIgE‐levels before or after CCD‐inhibition should be taken as clinically relevant.

Our results demonstrate that the CCD‐inhibition test is useful in patients who tested negative for recombinant honeybee allergens. Our patients, who had persistent HBV‐positivity even after CCD‐inhibition, require CRD to other recombinant honeybee allergens.

Molecular diagnostics is a major step toward personalized medicine,^45^ especially in patients who are exclusively sensitized to allergens that are underrepresented in therapeutic mixtures for VIT, as described for rApi m 10.^46^ We demonstrated that Immunoblot, using profile of the five most important recombinant allergens had a high sensitivity for diagnosis HVA. Patients with multiple HBV/WV/HV/CCD‐positivity had more frequently severe SAR.

In patients with multiple‐positivity, CCD‐inhibition increases sensitivity of CRD and help to define HBV allergic patients who require an extended CRD. CCD‐inhibition and CRD help synergistically to reveal the clinical relevant, true sensitization and improve the selection of allergens for long‐lasting VIT.

## AUTHOR CONTRIBUTIONS

**Dragana Jovanovic**: conceptualization (equal); data curation (equal); investigation (equal); methodology (equal); writing – original draft (equal). **Aleksandra Peric‐Popadic**: investigation (equal); validation (equal). **Vojislav Djuric**: data curation (equal); writing – review and editing (equal). **Maja Stojanovic**: data curation (equal); validation (equal); writing – review and editing (equal). **Branislav Lekic**: methodology (equal); software (equal). **Ognjen Milicevic**: software (equal). **Branka Bonaci‐Nikolic**: conceptualization (equal); investigation (equal); methodology (equal); supervision (equal); writing – review and editing (equal).

## CONFLICT OF INTEREST STATEMENT

The authors declare no conflicts of interest.

## Data Availability

The data that support the findings of this study are available from the corresponding author upon reasonable request.
